# Protocol for differentiating hematopoietic progenitor cells from human pluripotent stem cells in chemically defined monolayer culture

**DOI:** 10.1016/j.xpro.2024.103545

**Published:** 2025-01-11

**Authors:** Shaokang Mo, Kengyuan Qu, Jun Shen, Kuangyu Yen

**Affiliations:** 1 State Key Laboratory of Experimental Hematology, National Clinical Research Center for Blood Diseases, Haihe Laboratory of Cell Ecosystem, Institute of Hematology & Blood Diseases Hospital, Chinese Academy of Medical Sciences & Peking Union Medical College, Tianjin 300020, China

**Keywords:** cell biology, cell culture, developmental biology, stem cells, cell differentiation

## Abstract

Human pluripotent stem cells (hPSCs) provide a powerful platform for generating hematopoietic progenitor cells (HPCs) and investigating hematopoietic development. Here, we present a protocol for maintaining hPSCs and inducing their differentiation into HPCs through the endothelial-to-hematopoietic transition (EHT) on vitronectin-coated plates. We outline steps for evaluating the efficiency of HPC generation and assessing their potential to differentiate into various hematopoietic lineages. This protocol serves as a framework for exploring human hematopoiesis and generating various functional blood cells.

For complete details on the use and execution of this protocol, please refer to Shen et al.^1^ and Qu et al.^2^

## Highlights

• Generate multilineage hematopoietic progenitors from hPSCs in a defined monolayer culture
• Model the endothelial-to-hematopoietic transition from hPSCs under *in vitro* conditions
• Enhance HPC yield through intermediate isolation of hemogenic endothelial cells

## Abstract (editor highlights)

Publisher’s note: Undertaking any experimental protocol requires adherence to local institutional guidelines for laboratory safety and ethics.

## Graphical abstract

**Figure:**
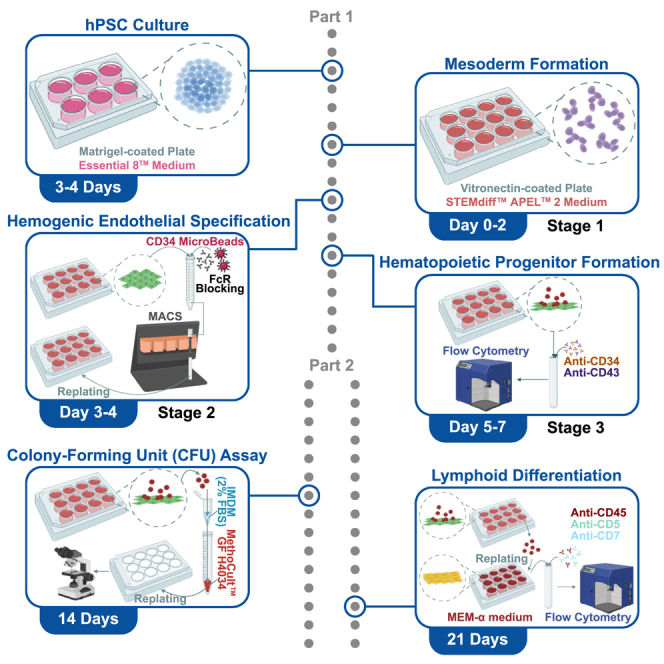

## Before you begin

### Institutional permissions

The human pluripotent stem cell line used in this study was obtained from the WiCell Research Institute (Madison, WI; http://www.wicell.org) with all necessary approvals. Approval from funding agencies and/or institution committees is required to replicate this protocol.

### Background

**Figure 1:**
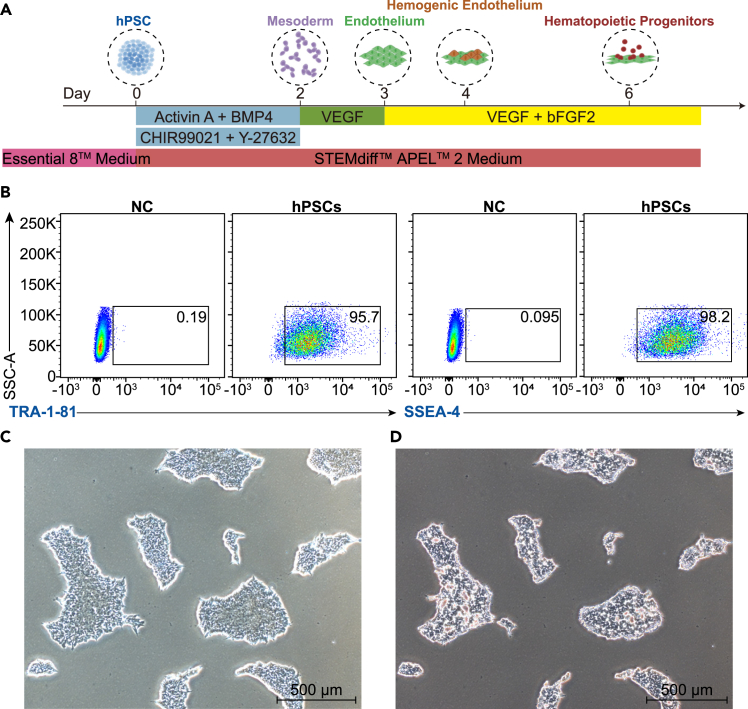
Characterization and preparation of hPSCs (A) Schematic representation of hematopoietic differentiation from hPSCs. (B) Flow cytometry analysis of hPSCs stained with TRA-1-81 and SSEA4 antibodies, with unstained hPSCs serving as the negative control (NC). (C) hPSCs at 70% confluence, displaying colony morphology suitable for differentiation (scale bar, 500 μm). (D) hPSCs treated with 0.5 mM EDTA for 3–4 min during passaging to obtain cell clusters (scale bar, 500 μm).

The molecular mechanisms governing the generation of human HPCs remain underexplored, partly due to inadequate availability of human embryonic material and associated ethical concerns. This protocol addresses these challenges by utilizing a serum-free, stroma-free monolayer culture system on vitronectin-coated plates, enabling the differentiation of hPSCs into HPCs through the EHT. Within this *in vitro* system, hPSCs (Day 0) progress stepwise from mesodermal (Day 2) to endothelial (Day 4) to hematopoietic lineages, ultimately generating HPCs with multilineage differentiation potential (Day 6) (Figure 1A). This protocol is structured into two main parts: (1) the **differentiation** of human pluripotent stem cells (hPSCs) into HPCs, and (2) the subsequent **verification and characterization** of these HPCs through phenotypic and functional assays. Together, these steps offer a robust platform for studying human hematopoiesis and generating functional blood cells.

### Differentiation part

This section of the protocol guides you through the step-by-step process of differentiating hPSCs into HPCs. It includes the preparation of essential reagents, the culture and passaging of hPSCs, and the induction of mesoderm formation, hemogenic endothelial specification, and hematopoietic progenitor formation. This part ensures that the differentiation process is carried out under optimal conditions to generate functional HPCs.

### Verification and characterization part

After differentiation, this section provides detailed approaches to evaluate the efficiency of HPC generation and their potential to differentiate into various hematopoietic lineages. Techniques such as flow cytometry and functional assays, including colony-forming unit (CFU) assays and lymphoid differentiation potential analysis, are used to confirm the successful generation of HPCs and assess their functionality.

### Preparation

#### Reagents and materials

Prepare all necessary reagents and materials in advance, including E8 medium, Matrigel-coated plates, and differentiation medium. Ensure that all equipment is calibrated and ready for both differentiation and verification stages.

#### Quality control of hPSCs

Before beginning the differentiation process, inspect hPSC colonies to ensure they meet the required criteria (e.g., compact colonies, 70% confluence, uniform size) to ensure reproducibility and efficiency in differentiation.

### Preparation of E8 medium

**Timing: 2 weeks before differentiation**

1. Thaw one vial (10 mL) of Essential 8 supplement at 4°C for at least 12 h.
2. Prepare the E8 medium.
  a. Add one vial of Essential 8 supplement to 500 mL of Essential 8 basal medium.
  b. Aliquot the mixture into 50 mL centrifuge tubes.
  c. Filter the mixture through a 0.22 μm filter to prevent bacterial contamination, and store at 4°C.

***Note:*** Once the supplement is mixed with the basal medium, it is referred to as the E8 medium.

### Preparation of Matrigel-coated 6-well plates

**Timing: 1 day before thawing hPSCs**

3. Thaw Matrigel (5 mL/vial) at 4°C for at least 12 h.
4. Mix 5 mL of Matrigel with 10 mL of pre-chilled E8 medium, aliquot 1 mL into pre-chilled 1.5 mL Eppendorf tubes, and store at −20°C.

***Note:*** This will yield 15 Eppendorf tubes.

5. Place one Eppendorf tube of mixed Matrigel on ice, dilute with 29 mL of pre-chilled E8 medium, and use the mixture to coat five 6-well plates (1 mL/well).

***Note:*** Antibiotics can be added to the E8 medium, but this is not required. Their use is mainly to prevent contamination during critical steps such as cell revival, media changes, or passaging. However, if these procedures are performed correctly under sterile conditions, contamination is generally unlikely.

6. Incubate the Matrigel-coated plates at +20°C for at least 30 min. After incubation, the plates can either be used immediately or stored at 4°C for up to one week.

***Note:*** If using the Matrigel-coated plates stored at 4°C, allow them to equilibrate to +20°C for at least 30 min before use.

7. Equilibrate the plates and E8 medium to +20°C for 30 min before use.

### Thawing and initial culture of hPSCs

**Timing: 4 days before differentiation (day 0 of hPSCculture)**

8. Quickly thaw frozen cryovials containing hPSCs in a 37°C water bath until a small amount of ice remains visible in the vial.
9. Transfer the cell suspension to a 15 mL tube containing 5 mL of E8 medium.
10. Centrifuge at 250 × *g* for 5 min.
11. Aspirate the supernatant and resuspend the pellet in 1 mL of E8 medium.
12. Carefully transfer the cell suspension to the Matrigel-coated 6-well plate, which contains 1 mL of E8+Y medium (E8 medium supplemented with 1 μL of 10 mM ROCK inhibitor (Y-27632)) per well.

***Note:*** Normally, hPSCs harvested from one well of a 6-well plate result in approximately 1×10^6^ cells per cryovial. However, it's important to note that the final cell count depends on the initial number of viable cells seeded per well. For instance, starting with 10^4^ viable cells per well can yield around 10^6^ cells after 2 days of culture. The optimal cell density for passaging or freezing is around 70%, with a cell count of approximately 10^6^ cells. After thawing, cell viability typically ranges from 60% to 70%. Therefore, the most suitable number of viable cells to seed per well is around 10^5^, equivalent to 1/10 of a 1 mL cell suspension, which results in 100 μL per well. To account for variability in cell viability and number after each freeze-thaw cycle, a concentration gradient of 100 μL, 200 μL, and 300 μL of cell suspension is recommended. During routine cell revival or passaging, the exact number of cells does not need to be extremely precise, and slight adjustments can be made based on these standard volumes.

13. Gently rock the plate back and forth to ensure uniform distribution.
14. Incubate the plates at 37°C in an incubator with 5% CO_2_.

***Note:*** This marks Day 0 of hPSCs culture. After 24 h of incubation, the hPSCs should be well-adhered and ready for medium replacement.

### Culture of hPSCs

**Timing: Day 1, and then daily from day 2 to day 3 or 4**

15. Aspirate the original medium and replace it with 2 mL of fresh E8 medium.

***Note:*** This step ensures the complete removal of Y-27632. This should be done near, but not beyond, 24 h of incubation after Day 0.

16. Incubate the plates at 37°C in an incubator with 5% CO_2_.
17. For the next two to three days (Day 2 through Day 3 or 4), replace the medium daily with 2 mL of fresh E8 medium.

***Note:*** Continue this until the cells reach approximately 70% confluency, at which point they will be ready for passaging or differentiation. Inspect the hPSCs daily under a microscope to verify growth status and cell morphology, and check for differentiation and contamination. Regular flow cytometry analysis is required to monitor the expression of pluripotency markers (such as *TRA-1-81* and *SSEA4*). High-quality hPSCs should express these markers (Figure 1B).

### Passaging of hPSCs

**Timing: Day 3 or 4 of hPSC culture**

**CRITICAL:** After thawing, hPSCs should be passaged at least once before initiating hematopoietic differentiation experiments. We recommend starting the hematopoietic differentiation after 2–3 passages to ensure efficient and reproducible results. Cells are ready for hematopoietic differentiation after 2–3 passages if they exhibit the following characteristics (Figure 1C): 1. compact colonies with bright edges and without noticeable dead cells; 2. reaching 70% confluency; 3. uniform colony size.

18. Prepare EDTA dissociation.
  a. Adding 50 μL of 0.5 M EDTA to 50 mL of DPBS.
  b. Filter using a 0.22 μm filter and store at +20°C.

***Note:*** When passaging hPSCs with EDTA, cells are transferred as small clusters rather than single cells to better support growth and maintain morphology post-passage.

19. Prepare the Matrigel-coated plate by aspirating any excess Matrigel solution and adding 2 mL of E8+Y medium per well.

***Note:*** Allow the plate to sit at +20°C for at least 30 min.

20. For the plate containing the hPSCs to be passaged, aspirate the medium carefully. Then, add 1 mL of the prepared EDTA solution per well. Incubate at +20°C for 2–4 min.

***Note:*** At this stage, the cells are adherent, not suspended, so they will remain attached to the plate when removing the medium. Cells should appear round but still clustered together (Figure 1D). Avoid over-digesting with EDTA.

21. Aspirate the EDTA solution and immediately add 1 mL of E8+Y medium to each well. Gently pipette up and down to separate the cells into small clusters.

***Note:*** Avoid excessive pipetting to prevent bubble formation.

22. Set up three gradients for passaging by transferring specific volumes of the cell suspension (e.g., 60 μL, 80 μL, and 100 μL) into the wells of the freshly prepared Matrigel-coated plate.

***Note:*** These volumes correspond to different cell seeding densities: 1:16, 1:12.5, and 1:10 dilutions, respectively, to determine optimal cell density for future differentiation experiments.

23. Gently swirl the plate back and forth and side to side to evenly distribute the cells.
24. Incubate at 37°C in an incubator with 5% CO_2_ to allow the cells to adhere.
25. Repeat the “culture of hPSCs” section.

## Key resources table

**Table:** 

REAGENT or RESOURCE	SOURCE	IDENTIFIER
**Antibodies**		

PE anti-human CD34 antibody, clone 581 (1:100)	BioLegend	Cat# 343506; RRID: AB_1731862
APC anti-human CD34 antibody, clone 561 (1:100)	BioLegend	Cat# 343608; RRID: AB_2228972
APC anti-human CD43 antibody, clone CD43-10G7 (1:100)	BioLegend	Cat# 343206; RRID: AB_2194072
PE anti-human CD43 antibody, clone CD43-10G7 (1:100)	BioLegend	Cat# 343204; RRID: AB_2255209
APC/Cyanine7 anti-human CD45 antibody, clone HI30 (1:200)	BioLegend	Cat# 304014; RRID: AB_314402
APC anti-human CD7 antibody, clone CD7-6B7 (1:200)	BioLegend	Cat# 343108; RRID: AB_2291325
PE/Cyanine7 anti-human CD5 antibody, clone UCHT2 (1:200)	BioLegend	Cat# 300622; RRID: AB_2275812
BD Pharmingen Alexa Fluor 555 mouse anti-SSEA-4, clone MC813-70 (1:100)	BD Biosciences	Cat# 560218; RRID: AB_1645389
TRA-1-81 (Podocalyxin) monoclonal antibody, APC, clone TRA-1-81 (1:100)	Invitrogen	Cat# 17-8883-42; RRID: AB_10597905
AO/PI fluorescent staining solution (1:1)	Countstar	Cat# RE010213
LS column	Miltenyi Biotec	Cat# 130-122-729
MACS MultiStand	Miltenyi Biotec	Cat# 130-042-303
Costar 6-well clear TC-treated multiple well plates, individually wrapped, sterile	Corning	Cat# 3516
Costar 12-well clear TC-treated multiple well plates, individually wrapped, sterile	Corning	Cat# 3513
12-well clear flat bottom ultra-low attachment plates, individually wrapped, sterile, skin packing	GeneBio Systems	Cat# 11218
Falcon 5 mL round bottom polystyrene test tube, with cell strainer snap cap	Corning	Cat# 352235

**Chemicals, peptides, and recombinant proteins**		

Essential 8 medium	Gibco	Cat# A1517001
STEMdiff APEL 2 medium	STEMCELL Technologies	Cat# 05275
MethoCult H4034 Optimum	STEMCELL Technologies	Cat# H4034
MEM α, no nucleosides	Gibco	Cat# 12561056
DPBS, no calcium, no magnesium	Gibco	Cat# 14190-144
TrypLE select enzyme (1X), no phenol red	Gibco	Cat# 12563-029
UltraPure 0.5 M EDTA	Invitrogen	Cat# 15575-038
Corning Matrigel hESC-qualified matrix, LDEV-free, 5 mL	Corning	Cat# 354277
Animal-free recombinant human vitronectin	PeproTech	Cat# AF-140-09
Stemolecule Y27632	Stemgent	Cat# 04-0012-10
D-(+)-Trehalose dihydrate	Sigma-Aldrich	Cat# T9531-500G
CHIR99021	ABM Inc	Cat# G611
Fetal bovine serum (FBS), qualified, Australia	Gibco	Cat# 10099141
Penicillin-streptomycin (P/S, AA)	Gibco	Cat# 15140122
DEPC-treated water	Invitrogen	Cat# AM9906
Animal-free recombinant human Activin A	PeproTech	Cat# AF-120-14E
Animal-free recombinant human BMP-4	PeproTech	Cat# AF-120-05ET
Recombinant human VEGF	PeproTech	Cat# 100-20
Recombinant human FGF2	ABM Inc	Cat# Z101457
Animal-free recombinant human SCF	PeproTech	Cat# AF-300-07
Animal-free recombinant human TPO	PeproTech	Cat# AF-300-18
Animal-free recombinant human Flt3-ligand	PeproTech	Cat# AF-300-19
Animal-free recombinant human IL-7	PeproTech	Cat# AF-200-07

**Critical commercial assays**		

CD34 MicroBeads, human (1:10)	Miltenyi Biotec	Cat# 130-046-702
FcR blocking reagent, human (1:10)	Miltenyi Biotec	Cat# 130-059-901

**Experimental models: Cell lines**		

BC1-T hPSC	Shen et al.^1^	N/A
H1 hPSC	WiCell	http://www.wicell.org
OP9-hDLL1	Shen et al.^3^	N/A

**Software and algorithms**		

GraphPad Prism 8	GraphPad Prism Inc	https://www.graphpad.com/
FlowJo v.10	BD Biosciences	https://www.flowjo.com/
Adobe Illustrator	Adobe	https://www.adobe.com/cn/
BioRender	Institution subscription	https://www.biorender.com

## Materials and equipment

**Table:** Hematopoietic differentiation medium (HDM)

Reagent	Final concentration	Amount
STEMdiff APEL2 Medium	N/A	500 mL
PFHM-II Protein-Free Hybridoma Medium	N/A	25 mL
**Total**	**N/A**	**525 mL**

Store at 4°C and keep for a maximum of 2–3 weeks.

**Table:** PBE buffer

Reagent	Final concentration	Amount
DPBS	N/A	48.95 mL
FBS	N/A	1 mL
EDTA	N/A	0.05 mL
**Total**	**N/A**	**50 mL**

Store at 4°C and keep for a maximum of 4 weeks.

**Table:** 5% D-(+)-Trehalose dihydrate solution

Reagent	Final concentration	Amount
DPBS	N/A	500 mL
D-(+)-Trehalose dihydrate	N/A	25 mg
**Total**	**N/A**	**500 mL**

Filter through a 0.22 μm filter and store at 4°C for up to 3 months.

**Table:** Hematopoietic differentiation cytokines and compounds

Reagent	Final concentration	Reconstitution
Activin A	4 ng/mL	10 μg/mL
BMP4	10 ng/mL	10 μg/mL
CHIR-99021	3 μM	10 mM
Y-27632	10 μM	10 mM
VEGF	40 ng/mL	40 μg/mL
bFGF	40 ng/mL	40 μg/mL

Aliquot 50 μL into a 1.5 mL microcentrifuge tube and store at −80°C for a maximum of 6 months. Limit freezing and thawing cycles.

**Table:** Mesoderm formation (Stage 1) medium

Reagent	Final concentration	Amount
Hematopoietic Differentiation Medium (HDM)	N/A	49.865 mL
Activin A (10 μg/mL)	4 ng/mL	0.02 mL
BMP4 (10 μg/mL)	10 ng/mL	0.05 mL
CHIR-99021 (10 mM)	3 μM	0.015 mL
Y-27632 (10 mM)	10 μM	0.05 mL
**Total**	**N/A**	**50 mL**

***Note:*** Calculate the required amount of Day 0 medium based on this ratio, and prepare it fresh for immediate use.

**Table:** Hemogenic endothelial specification (Stage 2) medium

Reagent	Final concentration	Amount
Hematopoietic Differentiation Medium (HDM)	N/A	0.999 mL
VEGF (40 μg/mL)	40 ng/mL	0.001 mL
**Total**	**N/A**	**1 mL**

***Note:*** Calculate the required amount of Day 2 medium based on this ratio, and prepare it fresh for immediate use.

**Table:** Hematopoietic progenitor formation (Stage 3) medium

Reagent	Final concentration	Amount
Hematopoietic Differentiation Medium (HDM)	N/A	0.998 mL
VEGF (40 μg/mL)	40 ng/mL	0.001 mL
bFGF (40 μg/mL)	40 ng/mL	0.001 mL
**Total**	**N/A**	**1 mL**

***Note:*** Calculate the required amount of Day 4 medium based on this ratio, and prepare it fresh for immediate use.

**Table:** Lymphoid differentiation cytokines

Reagent	Final concentration	Reconstitution
SCF	20 ng/mL	100 μg/mL
IL-7	5 or 20 ng/mL	100 μg/mL
Flt3-Ligand	5 or 20 ng/mL	100 μg/mL
TPO	5 ng/mL	100 μg/mL

Aliquot 20 μL into a 1.5 mL microcentrifuge tube and store at −80°C for a maximum of 6 months. Limit freezing and thawing cycles.

**Table:** Lymphoid differentiation (stage 1) medium

Reagent	Final concentration	Amount
MEM-α medium	N/A	7.995 mL
FBS	20%	2 mL
SCF (100 μg/mL)	20 ng/mL	0.002 mL
IL-7 (100 μg/mL)	5 ng/mL	0.0005 mL
Flt3-Ligand (100 μg/mL)	20 ng/mL	0.002 mL
TPO (100 μg/mL)	5 ng/mL	0.0005 mL
**Total**	**N/A**	**10 mL**

***Note:*** Calculate the required amount of lymphoid stage 1 medium based on this ratio, and prepare it fresh for immediate use.

**Table:** Lymphoid differentiation (stage 2) medium

Reagent	Final concentration	Amount
MEM-α medium	N/A	7.995.5 mL
FBS	20%	2 mL
SCF (100 μg/mL)	20 ng/mL	0.002 mL
IL-7 (100 μg/mL)	20 ng/mL	0.002 mL
Flt3-Ligand (100 μg/mL)	5 ng/mL	0.0005 mL
**Total**	**N/A**	**10 mL**

***Note:*** Calculate the required amount of lymphoid stage 2 medium based on this ratio, and prepare it fresh for immediate use.

### Step-by-step method details

Part 1: Differentiation of hPSCs into hematopoietic progenitor cells

**Figure 2:**
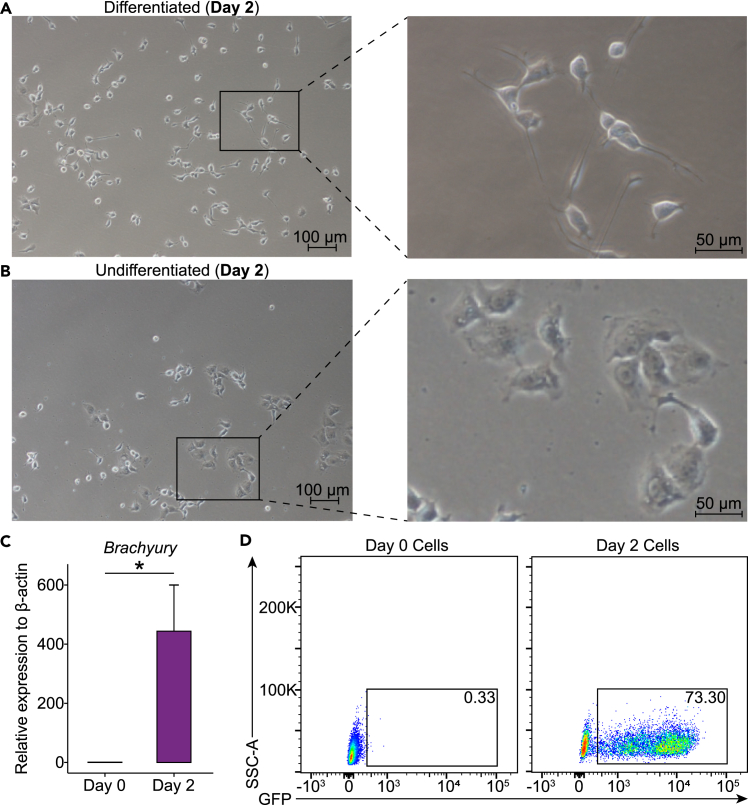
Morphological assessment of differentiation and undifferentiation states (A) Representative images of cells on day 2 of differentiation showing distinct morphological changes (left scale bar, 100 μm, right scale bar, 50 μm). (B) Representative image of undifferentiated cells on day 2, used as a control. Images were taken under the same conditions as in (A) (left scale bar, 100 μm, right scale bar, 50 μm). (C) Bar plot showing gene expression levels of Brachyury [T] at day 0 and day 2 differentiation. Data are presented as means ± SEM. ∗*p* < 0.05. (D) Flow cytometry analysis showing the expression level of Brachyury-conjugated GFP at Day 0 and Day 2 differentiation in BC1-T cells.

**Figure 3:**
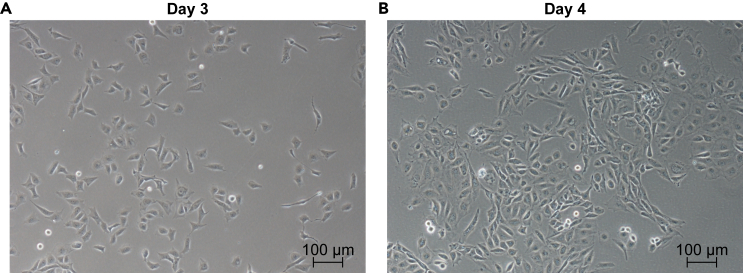
Morphological changes and hemogenic endothelial marker expression during differentiation (A) Morphology of differentiating cells on day 3 (scale bar, 100 μm). (B) Morphology of differentiating cells on day 4 (scale bar, 100 μm).

**Figure 4:**
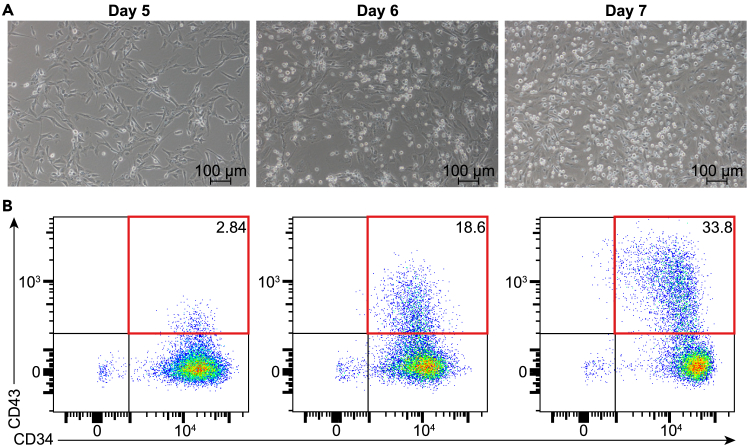
Hematopoietic Differentiation Progression from Days 5 to 7 (A) Morphological changes during hematopoietic differentiation on days 5, 6, and 7 (scale bar, 100 μm). (B) Flow cytometry analysis showing the expression of CD34 and CD43 on days 5, 6, and 7. Red compartment, CD34^+^CD43^+^ cells.

**Figure 5:**
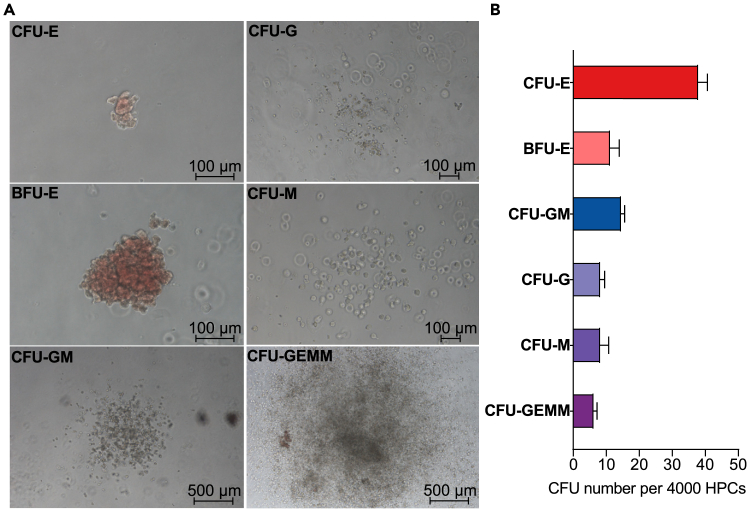
Colony-forming unit assay (A) Representative images of different colony types formed in the CFU assay: CFU-E (erythroid), CFU-G (granulocyte), BFU-E (burst-forming unit-erythroid), CFU-M (macrophage), CFU-GM (granulocyte-macrophage), and CFU-GEMM (granulocyte-erythroid-macrophage-megakaryocyte). The scale bar represents 100 μm for all images except CFU-GM and CFU-GEMM, which have a scale bar of 500 μm. (B) Quantification of CFU types per 4000 seeded cells. Data are presented as mean ± SEM from three independent experiments.

**Figure 6:**
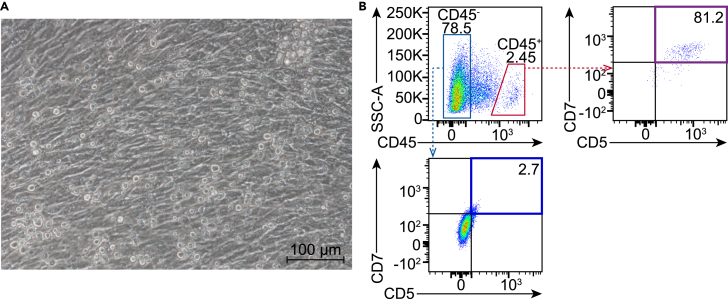
Lymphoid differentiation of hematopoietic progenitor cells (A) Morphology of cells undergoing lymphoid differentiation in co-culture with OP9-hDLL1 cells on day 21 (scale bar, 100 μm). (B) Flow cytometry analysis of lymphoid progenitor markers (CD5 and CD7) within the CD45^-^ (light blue) and CD45^+^ (red) compartments. Purple/deep blue compartments, CD5^+^CD7^+^ cells.

#### Mesoderm formation (stage 1)

**Timing: Approximately 2 h on day 0 for the initial setup, followed by a 48-h incubation period (days 0–2)**

***Note:*** This step involves inducing mesoderm formation, which is critical for the efficient generation of hemogenic endothelial cells and hematopoietic progenitor cells.

1. Preparation.
  a. Examine the hPSC colonies under a microscope to ensure they meet the required criteria for differentiation (e.g., compact colonies with bright edges, 70% confluency).
  b. Prepare a Vitronectin-coated 12-well plate by diluting Vitronectin solution with 5% D-(+)-Trehalose dihydrate solution at a 1:2 ratio (e.g., add 2 mL of 5% Trehalose solution to 1 mL of Vitronectin solution).
  c. Coat the 12-well plate with the diluted Vitronectin solution (0.5 mL per well) and incubate at +20°C for at least 1 h.
2. Induction.
  a. Aspirate the Vitronectin solution from the 12-well plate and immediately add 0.8 mL of freshly prepared mesoderm formation (Stage 1) medium per well along the wall of the well.
    ***Note:*** Ensure the plate does not dry out during this process.
  b. Aspirate the culture medium from the Matrigel-coated 6-well plate where the hPSCs have been cultured (as described in the “passaging of hPSCs” Section).
  c. Add 1 mL of 1X TrypLE Select Enzyme per well.
    ***Note:*** Observe under a microscope at +20°C. The digestion should take approximately 2 min. Proceed to the next step when cells appear rounded and translucent but have not yet fully detached.
  d. Gently aspirate the digestion solution, add 1 mL of mesoderm formation (stage 1) medium, and gently pipette up and down to resuspend the cells.
    ***Note:*** Ensure cells are fully dispersed as single cells in the suspension.
  e. Count the cells and seed them at a density of 6×10^3^ cells per well in the Vitronectin-coated 12-well plate containing mesoderm formation (stage 1) medium.
    ***Note:*** Seeding 6×10^3^ cells per well in a Vitronectin-coated 12-well plate is optimal for subsequent differentiation. Adjust as needed based on the specific hPSC line used.
  f. Gently shake the plate in all directions to evenly distribute the cells.
  g. Incubate the Vitronectin-coated 12-well plate at 37°C in an incubator with 5% CO_2_ for 2 days (48 h).
    **CRITICAL:** Observe the state of mesoderm differentiation under a microscope on Day 2, as this time point directly impacts the production of hematopoietic progenitor cells. Well-differentiated cells will be adherent with a full and translucent center and clear edges (Figure 2A). Undifferentiated cells are presented as dark, flattened cells (Figure 2B). Additionally, assess mesodermal gene expression (e.g., Brachyury [T]) to confirm differentiation (Figure 2C). Generally, we observe that approximately 70% of the total cell population expresses Brachyury (Figure 2D), as demonstrated by the BC1-T cell line (which conjugates the Brachyury gene with GFP, as previously constructed; see Shen et al.^3^).

#### Hemogenic endothelial specification (stage 2)

**Timing: Approximately 1 h on day 2 for the initial setup, followed by a 24-h incubation period on days 2–3, and another 1 h on day 3 for cytokine supplementation, followed by a final 24-h incubation period (days 3–4)**

***Note:*** This stage is characterized by the emergence of hemogenic endothelial cells, which are bipotent progenitors capable of generating both hematopoietic and endothelial cells.

3. Induction.
  a. On Day 2, gently aspirate the mesoderm formation (stage 1) medium and add 1 mL of the freshly prepared Hemogenic Endothelial Specification (Stage 2) medium per well.
    **CRITICAL:** On Day 2, cells are not firmly adherent and can easily detach. Exercise caution when changing the medium.
  b. Incubate the cells at 37°C in an incubator with 5% CO_2_ for 24 h.
  c. On Day 3, add 1 μL of bFGF to each well to reach a final concentration of 40 ng/mL, without changing the medium.
    ***Note:*** On Day 3, cell proliferation accelerates, resulting in increased cell numbers. Cells will appear darker, more flattened, and more adherent (Figure 3A).
  d. Incubate the cells at 37°C in an incubator with 5% CO_2_ for 24 h.

#### Hematopoietic progenitor formation (stage 3)

**Timing: Approximately 2–3 h on day 4 for the initial setup, followed by a 72-h incubation period (days 4–7)**

**CRITICAL:** By Day 4, the cell culture reaches approximately 90% confluency, and the cells increase in size, adopting a spindle-shaped, endothelial-like morphology (Figure 3B). At this stage, hemogenic endothelial cells (HECs) begin to give rise to hematopoietic progenitor cells (HPCs) through the endothelial-to-hematopoietic transition (EHT).^1^^,^^3^^,^^4^ To maximize HPC yield, we optimized the original protocol^1^ by isolating HECs on Day 4 before inducing hematopoiesis. Specifically, CD34^+^CD144^+^CD73^−^CD184^−^ cells present on Day 4 are identified as HECs.^1^^,^^2^^,^^5^^,^^6^ At this point, almost all CD34^+^ cells express CD144,^2^^,^^6^ and nearly all CD34^+^CD144^+^ cells are CD73^−^CD184^−^.^2^^,^^6^ Therefore, we use CD34 MicroBeads (**see**key resources table) to enrich HECs.

4. Preparation.
  a. Prepare a Vitronectin-coated 12-well plate as described in “mesoderm formation” step 1. **Preparation.** This plate will be used for seeding hemogenic endothelial cells after isolation.
5. Induction.
  a. Isolate hemogenic endothelial cells using CD34 MicroBeads:
    i. Aspirate the Hemogenic Endothelial Specification (Stage 2) medium from the Vitronectin-coated 12-well plate prepared in the “ hemogenic endothelial specification (stage 2)” Section and add 0.5 mL of 1X TrypLE Select Enzyme per well.
    ii. Once the cells have digested to a translucent state, gently detach them using a pipette and transfer them to a 50 mL centrifuge tube.
    iii. Add an equal volume of PBE buffer to the 50 mL centrifuge tube containing Day 4 hemogenic endothelial cells.
      ***Note:*** This step is done to dilute the TrypLE digestion solution. The PBE buffer serves two purposes: it neutralizes the TrypLE enzyme, stopping its action so it no longer digests the cells, and it helps maintain the cells in a stable environment.
    iv. Determine the cell number using a fluorescence cell analyzer: Mix 20 μL of the cell suspension with 20 μL of AO/PI fluorescent staining solution. Load 20 μL of the mixture into the analyzer using the appropriate chip.
      ***Note:*** Live and dead cells are distinguished by emitting green and red fluorescence, respectively.
      ***Note:*** In the following steps, 10^7^ cells will be used as an example. For 10^7^ cells, use at least 10 μL of human CD34 MicroBeads UltraPure for labeling. If the cell count is different from 10^7^, adjust the microbead volume proportionally based on a ratio of 10 μL per 10^7^ cells.
    v. Centrifuge the cell suspension at 350 × *g* for 5 min. Aspirate the supernatant completely.
    vi. Resuspend the cell pellet in PBE buffer at a ratio of 80 μL per 10^7^ total cells.
      ***Note:*** Gently pipette up and down or vortex at low speed to thoroughly resuspend the cell pellet in the buffer.
    vii. Add 10 μL of FcR Blocking Reagent (1:10) for up to 10^7^ total cells.
      ***Note:*** This step is crucial to prevent the non-specific binding of antibodies to Fc receptors on the surface of cells by binding to these receptors, ensuring that only the specific CD34 antibodies on the MicroBeads interact with the target CD34^+^ cells, thereby improving the specificity and purity of the cell isolation process.
    viii. Add 10 μL of human CD34 MicroBeads UltraPure (1:10) per 10^7^ total cells.
      ***Note:*** Mix the suspension well to ensure uniform distribution of the beads among the cells.
    ix. Incubate the cell-bead mixture at 4°C for 30 min.
      **CRITICAL:** Ensure that the mixture is periodically gently agitated or mixed to keep the cells evenly suspended and in contact with the beads.
    x. Wash the cells by adding 5 mL of PBE buffer to the cell-bead mixture.
    xi. Centrifuge at 350 × *g* for 5 min.
    xii. Aspirate the supernatant completely without disturbing the cell pellet to remove unbound beads and excess reagents.
    xiii. Resuspend the cells in 1 mL of PBE buffer.
      ***Note:*** Ensure the cells are evenly suspended for subsequent magnetic separation.
    xiv. Place an LS column in the magnetic field of a MACS Separator and then rinse the LS column with 1 mL of PBE buffer.
    xv. Gently pipette the prepared cell suspension (from **step xiii**) onto the LS column.
      ***Note:*** Magnetically labeled cells will be retained within the column matrix, and uncaptured cells will flow through.
    xvi. Rinse the LS column three times with 1 mL of PBE buffer each time.
      ***Note:*** This step aims to thoroughly wash away any non-specifically adhered cells or debris to ensure only the magnetically captured cells remain.
    xvii. Carefully remove the LS column from the MACS Separator and place it onto a 15 mL centrifuge tube for elution of the magnetic captured cells.
    xviii. Add 5 mL of PBE buffer to the top of the LS column. Immediately push the plunger provided with the LS column into the column.
      ***Note:*** It will flush out the magnetically captured cells into the 15 mL centrifuge tube placed beneath it.
    xix. Centrifuge the eluted magnetically captured cell suspension at 350 × *g* for 5 min.
    xx. Aspirate the supernatant without disturbing the cell pellet. Resuspend the cell pellet in 1 mL of hematopoietic progenitor formation (stage 3) medium to support the growth and differentiation of the progenitor cells.
    xxi. Determine the cell number, following the procedure described in **step iv.**
  b. Aspirate the Vitronectin solution from the fresh 12-well plates prepared in **step 4. Preparation** and swiftly add 2 mL of hematopoietic progenitor formation (stage 3) medium per well along the wall of the well.
  c. Seed the isolated CD34^+^ hemogenic endothelial cells at a density of 1.25×10^5^ cells per well in the Vitronectin-coated 12-well plate containing hematopoietic progenitor formation (stage 3) medium.
  d. Gently shake the plate in all directions to evenly distribute the cells.
  e. Incubate the Vitronectin-coated 12-well plate at 37°C in an incubator with 5% CO_2_.
    ***Note:*** As hematopoietic commitment progresses, small numbers of floating HPCs begin to appear around Day 5. The quantity of these HPCs gradually increases by Day 6, and by Day 7, a significant number of floating cells can be observed (Figure 4A). HPCs from Days 5–7 of differentiation exhibit the CD34^+^CD43^+^ phenotype, with their frequency increasing over time (Figure 4B). As shown in Figure 4B, the frequency of HPCs ranges from 2% to 30%, largely depending on the differentiation efficiency of earlier days (as discussed in the “troubleshooting” section). Additionally, different cell lines exhibit varying differentiation efficiencies. Despite this variability, HPCs remain functional even at the lower end of the range. Therefore, confirming the CD34^+^CD43^+^ cell ratio during each differentiation program is essential, as it provides a foundation for subsequent studies by ensuring reproducibility and allowing adjustment of experimental designs based on this ratio.
6. Verification of successful differentiation.
  **CRITICAL:** After induction, it's critical to verify that the cells have indeed acquired the expected phenotype. CD34 is a marker retained by progenitor cells,^7^ while CD43 is commonly upregulated as cells transition to a more committed hematopoietic progenitor stage.^8^^,^^9^ Testing for CD34 and CD43 expression via flow cytometry allows us to assess the purity and efficiency of differentiation.
  a. Perform flow cytometry analysis to examine CD34 and CD43 expression:
    i. For non-adherent cells, collect the medium from each well of the Vitronectin-coated 12-well plate into a 15 mL centrifuge tube.
    ii. For adherent cells, add 0.5 mL of 1X TrypLE Select Enzyme per well and observe under a microscope at +20°C.
      ***Note:*** The digestion should take approximately 2 min. Once the cells transition from a spindle-shaped, adherent state to a rounded, translucent state, gently pipette to detach them and transfer the cells into the 15 mL centrifuge tube containing the non-adherent cells.
    iii. Centrifuge the combined cell suspension at 350 × *g* for 5 min to pellet the cells.
    iv. Aspirate the supernatant.
  b. Resuspend the cell pellet in 1 mL of PBE buffer before centrifugate the cell suspension again at 350 × *g* for 5 min to wash away any residual reagents.
  c. After centrifugation, aspirate the supernatant, leaving a clean cell pellet ready for antibody staining.
  d. Under light-protected conditions, resuspend the cell pellet in 100 μL of PBE buffer.
  e. Add 1 μL of the CD34 antibody (1:100) and 1 μL of the CD43 antibody (1:100) to the cell suspension.
    ***Note:*** Similarly, add 1 μL of each antibody to the H1 hPSC cell line, which serves as a negative control since it does not express either CD34 or CD43.
  f. Mix the cell suspension thoroughly to ensure even antibody binding and incubate the cells at 4°C for 30 min.
  g. After incubation, add 1 mL of PBE buffer to the cell suspension.
  h. Centrifuge the cell suspension at 350 × *g* for 5 min to remove any unbound antibodies.
  i. Resuspend the cell pellet in 1 mL of PBE buffer.
  j. Transfer the cell suspension through the lid of the flow cytometry tube, which contains an integrated membrane.
    ***Note:*** This step filters the suspension, removing cell clumps or debris that could clog the flow cytometer, ensuring a smooth flow and accurate readings.
  k. Centrifuge the filtered cell suspension at 350 × *g* for 5 min to pellet the cells, then aspirate the supernatant.
  l. Resuspend the cell pellet in 100 μL of PBE buffer before adding 1× DAPI solution to the suspension.
    ***Note:*** This step gates out dead cells during flow cytometry analysis, ensuring accurate assessment of CD34 and CD43 expression in viable cells.
  m. Load the prepared cell suspension onto the flow cytometer to determine CD34 and CD43 levels.

### Part 2: Verification and characterization of hematopoietic progenitor cells

#### Colony-forming unit assay for functional characterization of hematopoietic progenitor cells

**Timing: Approximately 2 h on day 0 for the initial setup, followed by a 14-day incubation period**

***Note:*** The CFU assay is an *in vitro* functional method used to quantify multipotential and lineage-specific HPCs in various hematopoietic tissues. It enables the identification and classification of erythroid, granulocyte/macrophage, and multilineage progenitor cells, as each colony represents a unique progenitor cell.

7. Preparation.
  a. Retrieve the suspended cells from the “ hematopoietic progenitor formation (stage 3)” Section and prepare 4000 cells in 0.1 mL of IMDM containing 2% FBS.

***Note:*** In this protocol, we recommend using all the suspended cells instead of just marker-defined subpopulations (such as CD34^+^CD43^+^). The reason is to keep the process simple while still effectively showing that the HPCs have been successfully produced. Furthermore, in our experience, we found that whether we used all the suspended cells or just the CD34^+^CD43^+^ subpopulations, the number and types of colonies formed were similar. This suggests that most of the suspended cells are already the HPCs of interest. Hence, by including all the suspended cells, we simplify the process without losing any valuable information about the functionality of the HPCs.

**CRITICAL:** To ensure clear identification and counting of hematopoietic colonies, it is important to start with a low number of cells. For example, in a 12-well plate, aiming for 50–100 colonies per well is optimal. Because different experimental conditions might yield different numbers of hematopoietic progenitor cells (HPCs), it is recommended to first test various starting cell densities to find the best amount for your specific setup. In our hands, using this protocol, starting with 4000 cells per well has been identified as the optimal input number to achieve the desired range of colonies.

8. Induction.
  a. Mix the cell suspension with 2 mL of MethoCult H4034 Optimum.
  b. Dispense the mixture into ultra-low attachment 12-well plates.
  c. To prevent evaporation, fill the space around the wells with sterile ultrapure water.
  d. Incubate the plates at 37°C in an incubator with 5% CO_2_ for 14 days.
  e. After 14 days, classify each colony type based on its morphology and count the colonies.

***Note:*** Six distinct hematopoietic lineage colony types can be identified based on morphology and size, including Colony-Forming Unit-Erythroid (CFU-E), Burst-Forming Unit-Erythroid (BFU-E), Colony-Forming Unit-Granulocyte (CFU-G), Colony-Forming Unit-Macrophage (CFU-M), Colony-Forming Unit-Granulocyte, Macrophage (CFU-GM), and Colony-Forming Unit-Granulocyte, Erythroid, Macrophage, Megakaryocyte (CFU-GEMM) (Figures 5A and 5B). The results of the CFU assay confirm the differentiation and proliferation potential of the HPCs.

#### HPCs functional characterization for lymphoid differentiation potential

**Timing: Approximately 1 h every 4 days over a 21-day period**

***Note:*** Lymphoid differentiation potential is a crucial indicator for assessing the multilineage differentiation capacity of hematopoietic stem and progenitor cells (HSPCs). Differentiating HPCs into lymphoid progenitor cells (CD45^+^CD5^+^CD7^+^) allows for the identification of their lymphoid differentiation potential.

9. Preparation.
  a. Culture the OP9-hDLL1 cell line (previously constructed, refer to Shen et al.^3^) in MEM-α medium supplemented with 10% FBS.
  b. One day before starting the human lymphoid progenitor cell differentiation experiment, seed 1×10^5^ OP9-hDLL1 cells in ultra-low attachment 12-well plates.
10. Induction.
  a. On Day 0 of the lymphoid differentiation experiment, seed 1.5×10^5^ suspended HPCs from the “hematopoietic progenitor formation (stage 3)” Section onto the OP9-hDLL1 cells in the 12-well plate, using freshly prepared lymphoid differentiation stage 1 medium.
  b. Incubate the 12-well plate at 37°C in an incubator with 5% CO_2_ until Day 7.
    **CRITICAL:** Change the medium every 3–4 days. Prepare fresh OP9-hDLL1 feeder cells one day in advance by seeding 1×10^5^ OP9-hDLL1 cells in 12-well plates. During the medium change, collect the suspended hematopoietic cells, centrifuge at 350 × *g* for 5 min, aspirate the supernatant, and then reseed the cells onto the OP9-hDLL1 feeder layer with lymphoid stage 1 medium.
  c. On Day 7, switch to lymphoid differentiation stage 2 medium. Incubate the 12-well plate at 37°C in an incubator with 5% CO_2_ until Day 21. Change the medium every 3–4 days.
11. Characterization.
  a. On Day 21, perform flow cytometry to test for the expression of CD45, CD5, and CD7:
    i. Collect the supernatant into a 15 mL centrifuge tube.
    ii. Digest the adherent cells with 1X TrypLE Select Enzyme (no phenol red) for 3 min, then combine them with the suspended cells in the same 15 mL centrifuge tube.
    iii. Centrifuge at 350 × *g* for 5 min and aspirate the supernatant.
    iv. Resuspend 1×10^6^ cells in 1 mL of PBE buffer.
    v. Wash the cells by centrifugation at 350 × *g* for 5 min, and aspirate the supernatant.
    vi. Resuspend the cells in 200 μL of PBE buffer.
    vii. Under light-protected conditions, add the CD45, CD5, and CD7 antibodies (1:200), using an isotype control as a reference.
    viii. Thoroughly mix the suspension and incubate at 4°C for 30 min while avoiding light.
    ix. Wash the cells by adding 1 mL of PBE buffer and filter the cell suspension through a membrane.
    x. Centrifuge at 350 × *g* for 5 min, and aspirate the supernatant.
    xi. Resuspend the cells in 200 μL of PBE buffer.
    xii. Just before analysis, add 1× DAPI solution to differentiate live and dead cells.

***Note:*** During the later stages of differentiation, some suspended hematopoietic cells may adhere to the feeder layer (Figure 6A). To ensure complete collection of the suspended hematopoietic cells, we recommend digesting all cells for thorough analysis. On Day 21, lymphoid progenitor markers CD5 and CD7 should be predominantly expressed in the CD45^+^ subset (Figure 6B).

## Expected outcomes

This protocol provides an efficient and reliable method for generating functional hematopoietic progenitor cells (HPCs) from human pluripotent stem cells (hPSCs) through a carefully optimized stepwise differentiation process. The goal is to produce HPCs that are phenotypically identifiable and functionally competent, suitable for downstream research and potential therapeutic applications.

Throughout the differentiation process, hPSCs undergo mesodermal induction, hemogenic endothelial specification, and hematopoietic lineage commitment. By day 4, a significant population of cells expresses key markers such as CD34 and CD144,^2^^,^^6^ indicating their transition into hemogenic endothelial cells. As differentiation progresses, these cells will further develop into multipotent HPCs, characterized by the expression of both CD34 and CD43 (Figure 4B).

The protocol is designed to maximize the yield and functionality of the resulting HPCs. By day 7, the generated HPCs are expected to display a robust hematopoietic phenotype with the ability to differentiate into various blood lineages, including myeloid and lymphoid cells. The efficiency of this differentiation process can be assessed through CFU assays, where the HPCs demonstrate their capacity to form distinct hematopoietic colonies, including CFU-GEMM (granulocyte, erythrocyte, monocyte, megakaryocyte) (Figures 5A and 5B). Additionally, the HPCs generated in our *in vitro* system demonstrate strong lymphoid differentiation potential (Figures 6A and 6B).

The anticipated outcomes include a high yield of HPCs with consistent expression of CD34^+^CD43^+^ markers, reflecting their hematopoietic potential. These cells are expected to exhibit functional properties that allow them to contribute to hematopoiesis, making them valuable for both *in vitro* studies and potential *in vivo* applications.

Overall, this protocol offers a robust framework for generating HPCs that are functionally competent and suitable for various research and therapeutic purposes. The ability to produce a consistent and scalable population of HPCs from hPSCs under defined conditions ensures the reproducibility and reliability of this method across different hPSC lines and experimental setups.

## Limitations

While this protocol is designed to achieve high differentiation efficiency of HPCs from hPSCs, several factors can influence the overall yield and consistency of the results. Despite aiming for robustness and reproducibility, variability may still arise due to the genetic background and source of the hPSCs used. The differentiation potential of different hPSC lines can vary significantly, resulting in fluctuations in HPC yield. On average, this protocol generates HPCs by days 6–7 of differentiation; however, certain cell lines may exhibit lower efficiency due to inherent genetic differences.

Additionally, the process of mesodermal induction and hemogenic specification is highly sensitive to the initial conditions of hPSC culture, such as the size and morphology of the colonies before differentiation. Variations in these parameters, particularly during the mesodermal induction phase, can lead to inconsistent outcomes. For example, if the colonies are too large or too small, the efficiency of hematopoietic differentiation can be significantly affected.

Another potential limitation is the precise handling and timing of medium changes and cytokine additions, which are crucial for optimal differentiation. Small deviations in timing or concentrations can result in suboptimal differentiation, reducing the overall yield of functional HPCs.

## Troubleshooting

### Problem 1

Poor hPSC colony morphology (“culture of hPSCs” section).

### Potential solution

hPSCs are sensitive and typically require 2–3 passages post-thaw to regain stability. We recommend using hPSCs with a passage number of no more than 50 for hematopoietic differentiation. To ensure an adequate cell reserve, it is essential to cryopreserve early-passage cells.

### Problem 2

Previously successful cell lines fail to differentiate. By Day 2, microscopy reveals dark, flattened cells (“mesoderm formation (stage 1)” section)(Figure 2B).

### Potential solution

The quality of the differentiation medium (e.g., STEMdiff APEL 2) and cytokine stocks is critical for optimal outcomes. Store differentiation media and cytokine aliquots at −80°C, and thaw them at 4°C before use. Prepare small, single-use batches to avoid repeated freeze-thaw cycles, which can reduce efficacy. After thawing, store differentiation media and cytokines at 4°C, but use them within one week as their activity diminishes over time, potentially affecting differentiation potential.

### Problem 3

A high number of dead cells and uneven distribution of adherent cells were observed on Day 3 of differentiation (“hemogenic endothelial specification (stage 2)” section, step 3).

### Potential solution

On Day 2, cells are loosely adherent and prone to detachment. When adding D2 medium, position the pipette tip close to the well wall and bottom without touching them, and dispense the medium slowly. After changing the medium, avoid moving the plate to prevent cell detachment.

### Problem 4

During HE sorting on Day 4 differentiation (“hematopoietic progenitor formation (stage 3)” section step 5. a. ix), the proportion of CD34^+^ cells was relatively low.

### Potential solution

First, as noted in Problems 1–3, poor differentiation efficiency during the first three days may result in a low yield of HE on Day 4; therefore, ensure all differentiation steps are carefully followed. Second, during Day 3 differentiation, the amount of bFGF retained in the medium may be inconsistent. Change the culture medium with hematopoietic progenitor formation (stage 3) **medium** (see “materials and equipment”) if necessary. Last, when enriching HE with CD34 microbeads, CD34-positive cells may remain unlabeled if microbeads settle at the bottom of the tube. After the 10–15 min labeling period, inspect the tube for settled microbeads; if present, gently re-suspend the mixture to ensure even distribution and proper labeling of CD34-positive cells.

### Problem 5

Loss of HPCs during the medium change for lymphoid differentiation (“HPCs functional characterization for lymphoid differentiation potential” section, step 4).

### Potential solution

HPCs may adhere to stromal cells during medium changes for lymphoid differentiation. To minimize loss, we recommend partially digesting the cells to create a semi-adherent state, then gently pipette to collect HPCs while avoiding excess removal of stromal cells.

## Resource availability

### Lead contact

Further information and requests for resources and reagents should be directed to and will be fulfilled by the lead contact, Kuangyu Yen (kuangyuyen@ihcams.ac.cn).

### Technical contact

Questions about the technical specifics of performing the protocol should be directed to and will be answered by the technical contact, Jun Shen (shenjun@ihcams.ac.cn).

### Materials availability

This study did not generate new unique reagents.

### Data and code availability

This study did not generate or analyze any unique datasets.

## Acknowledgments

This work was supported by the National Key Research and Development Program of China (2018YFA0800201, 2021YFA1103000, and 2021YFA1100703), the National Natural Science Foundation of China (31872843, 31522031, and 82470230), the Science, Technology & Innovation Project of Xiongan New Area (2022XAGG0142), the Haihe Laboratory of Cell Ecosystem Innovation Fund (24HHXBSS00014), and the SKLEH-Pilot Research Grant (Z24-02). The graphical abstract was created using Biorender.com.

## Author contributions

K.Q. and S.M. performed the *in vitro* assays. S.M., K.Q., and K.Y. analyzed the results, made figures, and wrote the manuscript. K.Y. and J.S. commented on the manuscript.

## Declaration of interests

The authors declare no competing interests.

## References

[1] Shen J., Lyu C., Zhu Y., Feng Z., Zhang S., Hoyle D.L., Ji G., Brodsky R.A., Cheng T., Wang Z.Z. (2019). Defining early hematopoietic-fated primitive streak specification of human pluripotent stem cells by the orchestrated balance of Wnt, activin, and BMP signaling. J. Cell. Physiol..

[2] Qu K., Mo S., Huang J., Liu S., Zhang S., Shen J., Yen K. (2024). SPI1-KLF1/LYL1 axis regulates lineage commitment during endothelial-to-hematopoietic transition from human pluripotent stem cells. iScience.

[3] Shen J., Xu Y., Zhang S., Lyu S., Huo Y., Zhu Y., Tang K., Mou J., Li X., Hoyle D.L. (2021). Single-cell transcriptome of early hematopoiesis guides arterial endothelial-enhanced functional T cell generation from human PSCs. Sci. Adv..

[4] Shen J., Zhu Y., Zhang S., Lyu S., Lyu C., Feng Z., Hoyle D.L., Wang Z.Z., Cheng T. (2021). Vitronectin-activated alphavbeta3 and alphavbeta5 integrin signalling specifies haematopoietic fate in human pluripotent stem cells. Cell Prolif..

[5] Ditadi A., Sturgeon C.M., Tober J., Awong G., Kennedy M., Yzaguirre A.D., Azzola L., Ng E.S., Stanley E.G., French D.L. (2015). Human definitive haemogenic endothelium and arterial vascular endothelium represent distinct lineages. Nat. Cell Biol..

[6] Mo S., Qu K., Huang J., Li Q., Zhang W., Yen K. (2023). Cross-species transcriptomics reveals bifurcation point during the arterial-to-hemogenic transition. Commun. Biol..

[7] Sidney L.E., Branch M.J., Dunphy S.E., Dua H.S., Hopkinson A. (2014). Concise review: evidence for CD34 as a common marker for diverse progenitors. Stem Cell..

[8] Vodyanik M.A., Thomson J.A., Slukvin I.I. (2006). Leukosialin (CD43) defines hematopoietic progenitors in human embryonic stem cell differentiation cultures. Blood.

[9] Kessel K.U., Bluemke A., Schöler H.R., Zaehres H., Schlenke P., Dorn I. (2017). Emergence of CD43-Expressing Hematopoietic Progenitors from Human Induced Pluripotent Stem Cells. Transfus. Med. Hemother..

